# MicroRNA-27b up-regulated by human papillomavirus 16 E7 promotes proliferation and suppresses apoptosis by targeting polo-like kinase2 in cervical cancer

**DOI:** 10.18632/oncotarget.7531

**Published:** 2016-02-20

**Authors:** Fei Liu, Shimeng Zhang, Zhen Zhao, Xinru Mao, Jinlan Huang, Zixian Wu, Lei Zheng, Qian Wang

**Affiliations:** ^1^ Department of Laboratory Medicine, Nanfang Hospital, Southern Medical University, Guangzhou 510515, China; ^2^ Department of Clinical Laboratory, Guangzhou Women and Children's Medical Center, Guangzhou Medical University, Guangzhou 510623, China; ^3^ Central Laboratory, Shenzhen Shekou People's Hospital, Shenzhen 518000, China; ^4^ Department of Laboratory Medicine, NIH Clinical Center, Bethesda, MD 20892, USA

**Keywords:** microRNA-27b, HPV16 E7, cervical cancer, polo-like kinase2, DGCR8

## Abstract

The infection with high-risk human papillomavirus is linked to cervical cancer, nevertheless, the role of miRNAs regulated by HPV oncogenes in cancer progression remain largely unknown. Here, we knocked down endogenous E6/E7 in HPV16-positive CaSki cell lines, screened differences in miRNA expression profile with control using miRNA array. 38 miRNAs were down-regulated and 6 miRNAs were up-regulated in the E6/E7 silenced CaSki cells (>2-fold changes with *P* <0.05). The levels of miR-27b, miR-20a, miR-24, miR-93, and miR-106b were verified by qPCR in E6/E7 silenced CaSki and SiHa cells. MiR-27b, up-regulated by E7, promoted CaSki and SiHa cell proliferation and invasion, inhibit paclitaxel-induced apoptosis. Dual-luciferase experiment confirmed miR-27b down-regulated its target gene PLK2 through the “seed regions”. The tumor suppressor PLK2 inhibited SiHa cell proliferation, reduced cell viability, and promoted paclitaxel/cisplatin -induced apoptosis. Furthermore, DGCR8 was found to mediate the up-regulation of miR-27b by HPV16 E7. Our study demonstrated that HPV16 E7 could increase DGCR8 to promote the generation of miR-27b, which accelerated cell proliferation and inhibited paclitaxel-induced cell apoptosis through down-regulating PLK2. These findings provide an insight into the interaction network of viral oncogene, miR-27b and PLK2, and support the potential strategies using antisense nucleic acid of miR-27b for therapy of cervical cancer in the future.

## INTRODUCTION

Cervical cancer is one of the most malignant tumors. In 2012, 528,000 new cases of cervical cancer were diagnosed worldwide, of which 266,000 women died [1]. The primary cause of cervical cancer is persistent or chronic infection with one or more of the “high-risk” types of human papillomavirus(HPV) [2]. HPV is the most common sexually transmitted infection. Although vaccines to prevent HPV infections are available, WHO recommends routinely administering HPV vaccine to girls 9-13 years of age before sexual activity, as the preventive vaccines do not treat pre-existing HPV infection [1]. Moreover, it cannot yet be determined that the vaccines will work a lifetime. For patients with advanced-stage cervical cancer, there is no effective chemotherapy drugs, which make the search for novel therapeutic targets and the development of specific molecular targeting drugs important and urgent.

High-risk HPVs, such as HPV16 and HPV18, can be detected in 99.7% of cervical squamous cell carcinomas and 94-100% of cervical adeno and adenosquamous carcinomas [3, 4]. All malignant cells of HPV-positive cervical cancers contain at least one copy of the viral genome that is actively transcribed. The expression of two viral early oncoproteins E6 and E7 is an essential requirement for the development and maintenance of malignant phenotype.

Dysregulation of E6 and E7 expression increases genomic instability, accumulates genetic and epigenetic changes in cells. HPV16 E6 induces the ubiquitination and degradation of tumor suppressor protein p53, which maintains genomic integrity, and promotes apoptosis to prevent the variation of daughter cells [5]. HPV16 E7 can bind to and inactivate retinoblastoma protein(Rb), decompose Rb-E2F complexes through phosphorylation of Rb protein. The released E2F1 contributes to the trans-activation of c-Myc, cyclin A and E (cyclinA/E), in favor of intracellular DNA synthesis and cell transition from G1 to S phase, promoting cell proliferation [6–8].

Recent studies have shown that microRNAs (miRNAs) are transcriptional or post-transcriptional regulated by E6 and E7 [9]. MiRNAs are endogenous, non-coding RNAs with regulatory functions. The sizes of miRNAs are about 18 to 25 bases. By specific base pairing with the target mRNA, miRNAs can degrade, inhibit or activate target mRNA [10]. MiRNA itself, is transcriptionally regulated by transcription factors such as c-Myc, p53 and E2F, and post-transcriptionally regulated by proteins related to miRNA processing such as Drosha, DiGeorge Critical Region 8(DGCR8), and Dicer [11, 12]. The abnormal expression of miRNAs is discovered in most human tumors. MiRNAs can be involved in the process of tumorigenesis through regulating mRNA expression of oncogenes and tumor suppressor genes [13].

Although high-risk HPV has not its own miRNAs [14], it is proposed HPVs contribute to the up-regulation of host cell oncogenic miRNAs and decrease of tumor-suppressive miRNAs through comparisons between HPV positive and negative cell lines [15–17]. The ectopic expression of viral E6 and/or E7 genes in keratinocytes provides the direct evidence that viral oncogenes can influence the host cell miRNAs [18]. However, the effect of E6/E7-dependent miRNAs on maintenance of cell growth and invasion, or inhibition of apoptosis have not been demonstrated clearly.

In this study, we knocked down endogenous E6/E7 in HPV16-positive CaSki cell lines, screened differences in miRNA expression profile with control using miRNA array. A part of differentially expressed miRNAs was ascertained in both CaSki and SiHa cells by qPCR. The functions of miR-27b-3p (miR-27b) and its potential target gene on host cells phenotype were studied. Moreover, the mechanism of HPV16 E7 up-regulating miR-27b was investigated. These findings provide an insight into the interaction network of HPV oncogenes, miRNAs and target genes.

## RESULTS

### Down-regulation of HPV16 oncogene E6 and E7 by siRNA

**Figure 1:**
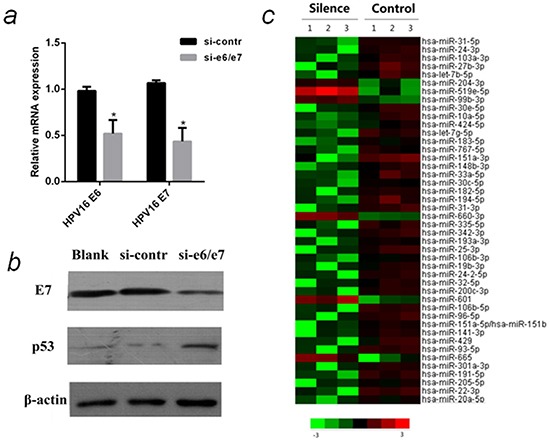
MiRNA array analysis of differentially expressed miRNAs in HPV16 E6/E7 silenced CaSki cells **a.** QPCR analysis of relative mRNA levels of HPV16 E6 and E7 in CaSki cells that were transfected with si-e6/e7 or si-contr. **b.** The protein levels of HPV16 E7 and p53 in CaSki cells that were transfected with si-e6/e7 or si-contr by western blot normalized to β-actin. **c.** Heat map representing the relative miRNA expression levels in HPV16 E6/E7 silenced CaSki cells(Silenced) and Control. The miRNAs (>2-fold changes with *P* < 0.05) were selected for hierarchical cluster analysis to generate the heat map. Each row represents a miRNA and each column represents a sample. The color bar depicts the color contrast level of the heat map. Red and green indicate high and low expression levels, respectively. *P < 0.05

In HPV16, E6 and E7 are transcribed as a single bicistronic E6E7 transcript using a common promoter and a common early polyadenylation site, thus one siRNA (si-e6/e7) is able to inhibit both E6 and E7 [19]. Compared with control, the mRNA levels of E6 and E7 were down-regulated in cells treated with si-e6/e7 (Figure 1a). Western bolt analysis showed E7 was suppressed, and p53 was up-regulated in cells transfected with si-e6/e7(Figure 1b). Since it is difficult to detect E6 protein, p53 protein was used as an indicator of E6 suppression because E6 can induce p53 degradation [19]. These results demonstrated E6 and E7 were efficiently down-regulated in si-e6/e7-transfected CaSki cells.

### MiRNA array analysis of differentially expressed miRNAs in HPV16 E6/E7 silenced cells

CaSki cell lines have a much higher copies (500∼600) of HPV16 than SiHa cell lines (1∼2), that is why we knock down E6/E7 in CaSki cells to discover differentially expressed miRNAs. MiRNAs of E6/E7-silenced CaSki cells were compared with control by miRNA array. 38 miRNAs were down-regulated and 6 miRNAs were up-regulated in the E6/E7 silenced cells (>2-fold changes with *P* <0.05). According to the fold change and literature search, five miRNAs [hsa-miR-27b-3p (miR-27b), hsa-miR-20a-5p (miR-20a), hsa-miR-24-3p (miR-24), hsa-miR-93-5p (miR-93), hsa-miR-106b-5p (miR-106b)] were selected for further analysis.

**Figure 2:**
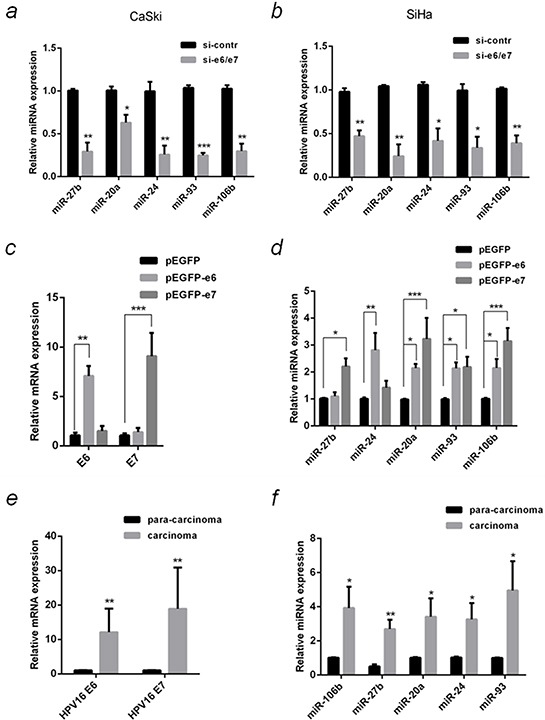
Verification of differentially expressed miRNA in HPV16(+) cervical cancer cells and tissues with qPCR **a.** The relative miRNAs levels in the samples same with microarray were detected by qPCR. **b.** The relative miRNAs levels in SiHa cells that were transfected with si-e6/e7 or si-contr were detected by qPCR. SiHa cells that were transfected with over-expression plasmids, pEGFP-e6, pEGFP-e7 or negative control (pEGFP), and the relative mRNA levels of HPV16 e6 and e7 **c.** or the selected miRNAs **d.** were detected by qPCR. **e.** HPV16 e6 and e7 mRNA in 10 pairs of HPV16(+) cervical cancer carcinoma and the corresponding tissue adjacent to carcinoma were detected by qPCR. **f.** The relative miRNAs in 10 pairs of HPV16(+) cervical cancer carcinoma and para-carcinoma were detected by qPCR. The 2^−ΔΔCT^ method was used for the relative quantification, with β-actin or U6 snRNA serve as endogenous controls for qPCR of mRNA or miRNA. The data are representative of three independent experiments.*P < 0.05, **P < 0.01, ***P < 0.001.

The differential expression of these five miRNAs was verified in E6/E7 silenced CaSki and SiHa cells using qPCR. The results confirmed that all of them were down-regulated in silenced group of both cell lines, consistent with the microarray results (Figure 2a & 2b).

### Differential miRNA expression in E6 and E7 over-expressed cells and in HPV16 (+) cervical cancer tissues

To understand which oncogene up-regulates these five miRNAs, plasmids over-expressing E6 and E7 (pEGFP-e6 and pEGFP-e7) were transfected into SiHa cells, respectively. Compared with control, E6 and E7 mRNA expression increased 7 and 9 folds, respectively (Figure 2c), meanwhile, the expression of the five miRNAs was increased (Figure 2d). MiR-20a, miR-93 and miR-106b were significantly up-regulated in E6 (by 2.1, 2.1 and 2.2 folds) or E7 (by 3.2, 2.2 and 3.2 folds) over-expressed cells. MiR-27b was up-regulated by 2.2 folds (*P* <0.05) in E7 but not E6 over-expressed cells. In contrary, E6 but not E7 increased miR-24 expression by 2.8 folds (*P* <0.01).

The expression of both E6 and E7 in HPV16 (+) cervical cancer tissues was significantly higher (*P* <0.01) than that in the paired adjacent normal tissues (Figure 2e). The five MiRNAs expression also increased significantly in carcinoma compared to paired para-carcinoma (Figure 2f).

### Function of miR-27b in cervical cancer on cell proliferation, invasion and apoptosis

Martinez [16] reported the level of miR-27b was increased in HPV16 positive CaSki cells in the comparison of HPV negative C33A cells, however, no further research was reported. Wang [20] reported miR-27b was abundant in cDNA clones of miRNAs expression profiles from cervical cancer-derived CaSki C-2 cells, where the isolation frequency of miR-27b from 363 cDNA clones was 8, little was known about the regulative role of miR-27b in cervical cancer yet. We also verified that the basal levels of miR-27b in CaSki and SiHa cells were also higher than HPV-negative C33A cells (Supplementary Figure S1). Besides, our microarray results showed the reduction of miR-27b was in the second place in E6/E7 silenced group (Supplementary Table S1). Therefore, we chose miR-27b to study further.

**Figure 3:**
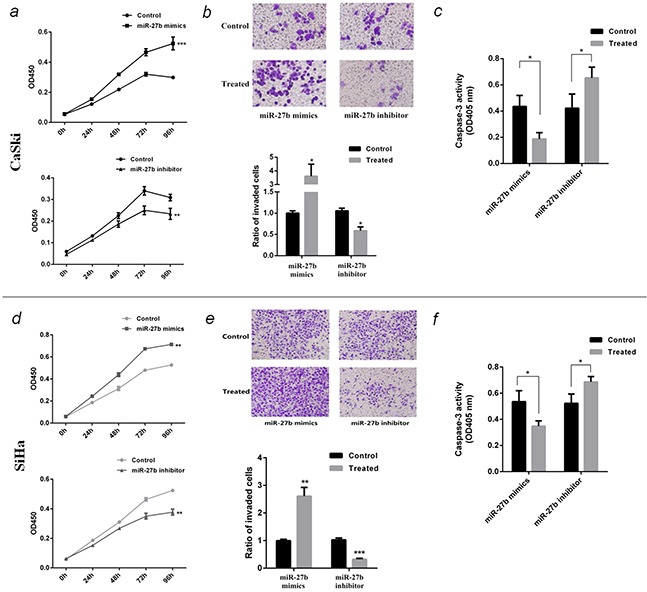
Function of miR-27b in CaSki and SiHa cells **a.** and **d.** The proliferation curve of transfected CaSki or SiHa cells were detected by CCK-8 test at OD450nm. **b.** and **e.** The invasion ability of transfected CaSki or SiHa cells were detected by transwell assay. The photograph showed the cells through the transwell chambers, stained by crystal violet. The histogram showed the ratio of invading cells compared to control. **c.** and **f.** The effect of miR-27b on paclitaxel-induced apoptosis on CaSki or SiHa cells were detected by caspase-3 activity assay at OD405nm. All the data are representative of three independent experiments. *P < 0.05, **P < 0.01, ***P < 0.001.

Over-expression of miR-27b promoted CaSki cell proliferation by 1.9 folds (*P* <0.001, Figure 3a), increased cell invasion by 3.6 folds (*P* <0.05, Figure 3b), and inhibited paclitaxel-induced apoptosis by 57% (*P* <0.05, Figure 3c). In contrary, inhibition of miR-27b restrained cell growth by 25% (*P* <0.01, Figure 3a), hampered cell invasion by 41% (*P* <0.05, Figure 3b) and accelerated paclitaxel-induced cell apoptosis by 1.6 folds (*P* <0.05, Figure 3c).

Similarly, the experiment in SiHa cells also showed ectopic expression of miR-27b promoted cell proliferation (*P* <0.01, Figure 3d), invasion (*P* <0.01, Figure 3e), and inhibited paclitaxel-induced apoptosis (*P* <0.05, Figure 3f), conversely, reducing miR-27b inhibited cell growth (*P* <0.01, Figure 3d), invasion (*P* <0.001, Figure 3e), and accelerate paclitaxel-induced cell apoptosis (*P* <0.05, Figure 3f).

**Figure 4:**
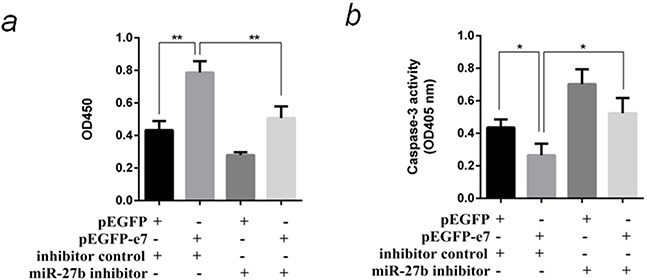
MiR-27b was responsible for phenotype resembling E7 functions in SiHa cells **a.** Cotransfect pEGFP-e7 and miR-27b inhibitor into SiHa cells, and detect the cell proliferation by CCK-8 test at OD450nm after 96 hours. **b.** Cotransfect pEGFP-e7 and miR-27b inhibitor into SiHa cells, and detect paclitaxel-induced apoptosis by caspase-3 activity assay at OD405nm. All the data are representative of three independent experiments. *P < 0.05, **P < 0.01.

To prove miR-27b is involved in E7 functions on change of host cells phenotype, a compensation experiment was designed. The plasmid pEGFP-e7 and miR-27b inhibitor were cotransfected into SiHa cells, then cell proliferation and paclitaxel-induced apoptosis were detected. The results indicated E7 promoted cell proliferation by 1.8 folds (*P* <0.01), inhibited cell apoptosis by 40% (*P* <0.05). In case of suppressing miR-27b at the same time, the above phenotype changes caused by E7 were partially counteracted (Figure 4a & Figure 4b).

### Prediction and verification of PLK2 as a miR-27b target

**Figure 5:**
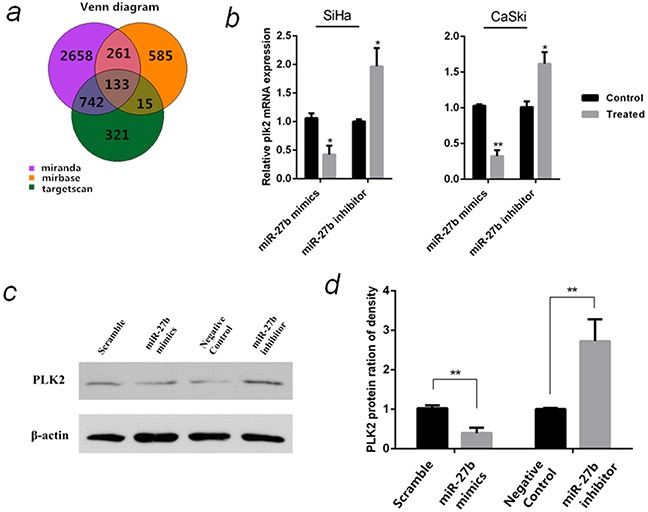
Prediction and verification of miR-27b target gene **a.** Prediction of miR-27b target genes by Targetscan, Microcosm and Miranda. **b.** The relative PLK2 mRNA levels in SiHa and CaSki cells that were transfected with miR-27b mimics or inhibitor were detected by qPCR, β-actin serves as endogenous controls. **c.** The protein levels of PLK2 in SiHa cells that were transfected with miR-27b mimics or inhibitor were detected by western blot normalized to β-actin. **d.** Statistical analysis of the PLK2 protein expression in the SiHa cells. The data are representative of three independent experiments. *P < 0.05, **P < 0.01.

To understand how miR-27b contributes to carcinogenesis of cervical cancer, its target genes were predicted using three online miRNA analysis database. 133 potential target genes were obtained from the intersection (Figure 5a), where four genes, PLK2 (polo-like kinase2) [21], PTCH1 [22], YEPL3 (Yippee-like 3) [23] and ZFP36 (ZFP36 ring finger protein) [24] were selected and their mRNA levels were detected using qPCR in CaSki and SiHa cells transfected with miR-27b mimics or inhibitors (data not shown). PLK2 mRNA expression was stably down-regulated in miR-27b over-expressed SiHa (by 58%, *P* <0.05) and CaSki cells (by 68%, *P* <0.01). In contrary, PLK2 mRNA expression was stably increased in miR-27b inhibited SiHa (2.0 folds, *P* <0.05) and CaSki cells (1.6 folds, *P* <0.05) (Figure 5b). The expression of PLK2 protein also changed inversely with miR-27b (Figure 5c & Figure 5d). These findings suggested that PLK2 might be a potential target gene of miR-27b.

**Figure 6:**
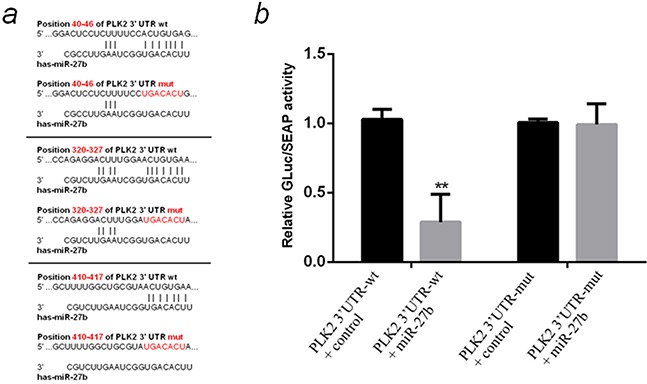
Prediction and verification of the “seed region” directly regulated by miR-27b on PLK2 mRNA 3′UTR **a.** Prediction of the “seed regions” directly regulated by miR-27b and the pattern of mutating the whole “seed regions”. All three “ACUGUGA” sites on 3′UTR of PLK2 mRNA were mutated to “UGACACU”. **b.** Detection of the relative GLuc/SEAP activity of miR-27b mimics cotransfected with PLK2 3′UTR-wt or PLK2 3′UTR-mut by dual-luciferase reporter test. All the data are representative of three independent experiments. **P < 0.01.

Subsequently, three databases were used to identify the “seed region” of miR-27b on PLK2 mRNA 3′UTR. All of the three databases predicted there are three potential miR-27b target regions on PLK2 mRNA 3′UTR (Figure 6a). To verify the direct effect of miR-27b on the “seed regions”, a wild-type PLK2 3′UTR (PLK2 3′UTR-wt) and a mutated PLK2 3′UTR with the whole three seed regions mutated(PLK2 3′UTR-mut) were chemically synthesized, and cloned into dual-luciferase reporter plasmids. The plasmids were cotransfected with control miRNA or miR-27b mimics. MiR-27b mimics significantly reduced the relative Gluc/SEAP activity when cotransfected with the PLK2 3′UTR-wt plasmid (by about 71%, *P* <0.01) but not the PLK2 3′UTR-mut (Figure 6b), which demonstrated miR-27b could work on the predicted “seed regions” to down-regulate the expression of PLK2.

### Function of PLK2 in cervical cancer on cell proliferation, invasion and apoptosis

**Figure 7:**
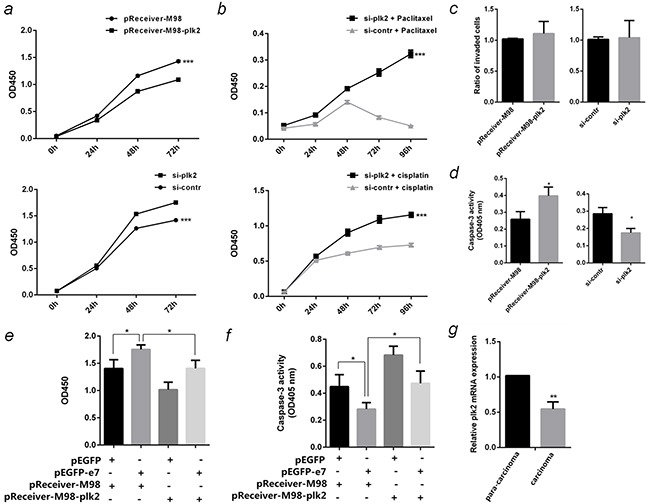
Function of PLK2 in SiHa cells **a.** The proliferation curve of PLK2 over-expressed or inhibited SiHa cells were detected by CCK-8 test at OD450nm. **b.** The effect of PLK2 on cell vitality after being treated with 1 μM paclitaxel or 2 μM cisplatin were detected by CCK-8 test. **c.** The effect of PLK2 on invasion ability of SiHa cells were detected by Transwell assay. **d.** The effect of PLK2 on paclitaxel-induced apoptosis in SiHa cells were detected by caspase-3 activity assay at OD405nm. **e.** Cotransfect pEGFP-e7 and pReceiver-M98-plk2 into SiHa cells, and detect the cell proliferation by CCK-8 test at OD450nm after 96 hours. **f.** Cotransfect pEGFP-e7 and pReceiver-M98-plk2 into SiHa cells, and detect paclitaxel-induced apoptosis by caspase-3 activity assay at OD405nm. **g.** PLK2 mRNA in 10 pairs of HPV16(+) cervical cancer carcinoma and the corresponding tissue adjacent to carcinoma were detected by qPCR. All the data are representative of three independent experiments. *P < 0.05, **P < 0.01, ***P < 0.001.

To determine the involvement of PLK2 in cervical cancer, functions of PLK2 on cell proliferation, invasion and paclitaxel-induced apoptosis were studied in PLK2 over-expressed or silenced SiHa cells. CCK-8 tests showed over-expression of PLK2 inhibited cell proliferation by 24% (*P* <0.001), while inhibition of PLK2 expression contributed to cell growth by 1.3 fold (*P* <0.001) (Figure 7a). When SiHa cells were transfected with si-plk2 and subsequently exposed to chemotherapy drug 1 μM paclitaxel or 2 μM cisplatin, the cells were more resistant and viable than control (*P* <0.001) (Figure 7b). Transwell assay indicated the invasion ability of SiHa cells cannot be influenced by PLK2 (Figure 7c). Caspase-3 activity tests showed over-expression of PLK2 speeded up paclitaxel-induced SiHa cell apoptosis by 1.5 folds (*P* <0.05), while the inhibition of PLK2 slowed down the apoptosis by 39% (*P* <0.05) (Figure 7d). To prove the carcinogenesis effect of E7 might be associated with down-regulation of PLK2, the plasmid pReceiver-M98-plk2 and pEGFP-e7 were cotransfected into SiHa cells, then cell proliferation and paclitaxel-induced apoptosis were detected. The results indicated the expression of PLK2 can counteract the effect of E7 on cell proliferation promoting and cell apoptosis inhibiting (Figure 7e & 7f). The expression of PLK2 in HPV16 (+) cervical cancer tissues was down-regulated compared to the paired para-carcinoma (*P* <0.01) (Figure 7g).

### HPV16 E7 up-regulated miR-27b through DGCR8

**Figure 8:**
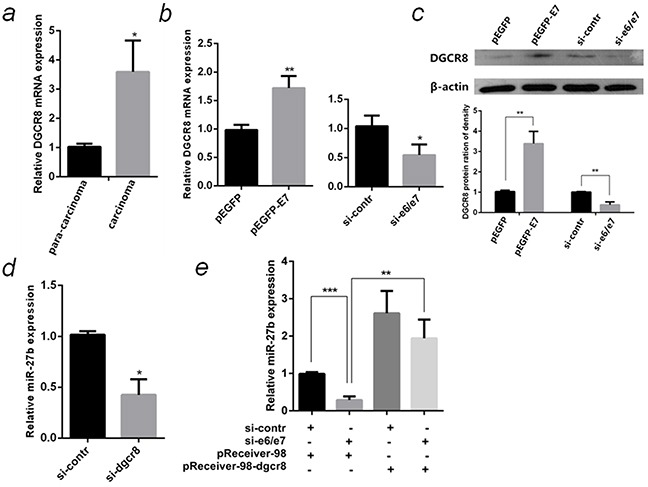
HPV16 E7 up-regulated miR-27b through DGCR8 **a.** The relative mRNA levels of HPV16 E7 and DGCR8 in 10 pairs of HPV16(+) cervical cancer carcinoma and para-carcinoma were detected by qPCR. **b.** The relative mRNA levels of DGCR8 in HPV16 E7 over-expressed or inhibited SiHa cells were detected by qPCR. **c.** The protein levels of DGCR8 in HPV16 E7 over-expressed or inhibited SiHa cells were detected by western blot normalized to β-actin. The histogram showed statistical analysis of the DGCR8 protein expression in the SiHa cells. **d.** The relative miR-27b level in DGCR8 silenced SiHa cells were detected by qPCR. **e.** The relative miR-27b level in SiHa cells cotransfected si-e6/e7 with the over-expression plasmid of DGCR8 were detected by qPCR. All the data are representative of three independent experiments. *P < 0.05, **P < 0.01, ***P < 0.001.

DGCR8, a double-stranded-RNA-binding protein, which combines with Drosha to form a microprocessor to generate miRNAs from the primary miRNA transcripts [25]. Recently, DGCR8 was reported to increase miR-27b in ovarian and breast cancer cells [26] [27]. This is an indication whether HPV16 E7 up-regulates miR-27b is mediated by DGCR8 in cervical cancer. The DGCR8 mRNA expression in carcinoma was significantly higher (3.5 Folds, *P* <0.05) than that in para-carcinoma, the changing trends are the same with HPV16 E7 (Figure 8a & Figure 2e). Both mRNA and protein levels of DGCR8 significantly increased in HPV16 E7 over-expressed SiHa cells, but reduced in E7-silenced cells (Figure 8b & 8c). MiR-27b was down-regulated by 70% (*P* <0.01) in DGCR8-silenced SiHa cells compared to control since miRNA processing was blocked (Figure 8d). When si-e6/e7 and the over-expression plasmid of DGCR8 were cotransfected into SiHa cells, DGCR8 was found to compensate for the reduction of miR-27b by E7 knockdown (Figure 8e).

## DISCUSSION

The incidence of cervical cancer is in the second place of gynecological oncology. Half of the patients die from the malignant tumor [28]. Surgery and radiotherapy are the main treatments for cervical cancer, however, surgery is only applicable to early stage, radiotherapy can cause irreversible damage to the ovaries and vaginas. Chemotherapy is only an adjuvant treatment to advanced cases, there is still not effective targeted therapy drugs [29]. Therefore, the research on molecular targeting drugs for cervical cancer treatment was greatly important.

Recent studies discovered that host cell miRNAs are dysregulated in high-risk HPV infected cervical cancer tissues and cells [20, 30]. Due to the significant carcinogenesis role of E6 and E7, Researchers have attempted to find whether the differentially expressed miRNAs were regulated by E6 or E7 [9, 31]. In this study, HPV16 E6/E7 were silenced in CaSki and SiHa cell lines, which both express wild-type p53 and Rb, to explore the differentially expressed miRNAs. MiR-27b was proved to be up-regulated by HPV16 E7 through DGCR8 to promote proliferation and suppresses apoptosis by targeting PLK2 in cervical cancer cells.

MiR-27b also increased in breast cancer [32] and glioma [33], related to cell growth, invasion and poor prognosis, otherwise decreased in colorectal cancer [34] and metastatic, castration-resistant prostate cancer [35] as tumor suppressor. Our studies showed that miR-27b promoted CaSki and SiHa cell proliferation and invasion, inhibited paclitaxel-induced apoptosis. The results indicated miR-27b played a cancer-promoting role in cervical cancer, in accordance with its role in breast cancer and glioma.

Knockdown of miR-27b augmented Nischarin (NISCH) and suppressor of tumorigenicity 14 (ST14) expression in human breast cancer [32, 36], down-regulated STAT3, c-myc and cyclin D1 in glioma [33]. To understand how miR-27b contribute to cervical carcinogenesis, its target genes PLK2 was discovered. MiR-27b was proved to down-regulate PLK2 through its “seed regions” on 3′UTR. This is the first report that PLK2 is regulated by miRNA in cervical cancer.

PLK2 belongs to polo-like kinases family, which is a serine/threonine protease family, mainly play a critical role in cell cycle and response to DNA damage [37]. In our studies PLK2 was found to inhibit cervical cancer cell proliferation, promote paclitaxel-induced apoptosis, and reduce cell viability when exposed to paclitaxel and cisplatin. The results indicated PLK2 play a tumor suppressive role in cervical cancer, similarly with its role in Burkitt lymphoma (BL) and epithelial ovarian cancer (EOC) [38] [39]. The aberrant cytosine methylation occurs in the CpG island of PLK2 gene's 5′end in BL and EOC, where PLK2 is transcriptionally down-regulated. Ectopic expression of PLK2 resulted in apoptosis in BL cells or restored sensitivity to G(2)-M cell-cycle blockade and cytotoxicity triggered by paclitaxel in EOC. In our studies, PLK2 was found to be regulated by miRNA-mRNA interaction. Whether other regulating mechanism to PLK2, like CpG island methylation, exsits in cervical cancer should be studied further.

Recently, Honegger [40] reported a worthwhile deep sequencing study which showed that E6/E7 silencing up-regulated miR-27b in HPV18-positive HeLa cells, and miR-27b was supposed to be anti-tumorigenic. It seems contradictory to our results. To understand the difference, it need to make the following considerations: (i) Different HPV-positive cervical cancer cells were used in these two E6/E7 silencing models. (ii) MiR-27b played a contradictory effect in many kinds of tumors. Our experiments in CaSki and SiHa cells indicate miR-27b played a cancer-promoting role in cervical cancer. This finding is consistent with the prospection that HPV oncogenes up-regulated oncogenic miRNAs to promote carcinogenesis. (iii) PLK2, the target gene of miR-27b, was discovered by miR-27b gain or loss experiments and verified by dual-luciferase report test. In addition, PLK2 was proved to be a tumor suppressor in SiHa cells. These findings were consistent with the oncogenic role of miR-27b in cervical cancer. (iv) The up-regulation of miR-27b and down-regulation of PLK2 were confirmed in HPV16 positive cervical cancer tissues. Therefore, the evidences above support miR-27b as a cancer-promoting factor to be up-regulated by HPV oncogene in cervical cancer.

The role of miR-27b in cervical cancer is only beginning to be elucidated. The fact that miR-27b is up-regulated by virus oncogene E7 through DGCR8, negatively regulates PLK2 in HPV16 positive cervical cancer cells suggests that miR-27b is likely to play an oncogenic role in cervical cancer to promote cell proliferation and inhibit paclitaxel-induced cell apoptosis. This study is one step forward in understanding miR-27b-dependent regulation, provides an insight into the interaction network of viral oncogene, miR-27b and PLK2, and supports the potential strategies using miR-27b-targeted antisense nucleic acid for therapy of cervical cancer in the future.

## MATERIALS AND METHODS

### Plasmids, siRNA and primers

HPV 16 e6 and e7 ORFs were cloned into pEGFP-C3 expression vectors, and the resulting plasmids were designated as pEGFP-e6 and pEGFP-e7, respectively(Land, Guangzhou, China). PLK2 and DGCR8 over-expression plasmid, pReceiver-M98-plk2 and pReceiver-M98-dgcr8, were purchased from GeneCopoeia (Guangzhou, China). SiRNA anti-hpv16 e6/e7 (si-e6/e7) [19], anti-plk2(si-plk2) and anti-dgcr8 (si-dgcr8), control siRNA (si-contr), miR-27b mimics and inhibitors were synthesized (Ribobio, Guangzhou, China). Real-time quantitative PCR (qPCR) primers used for examining target genes and internal reference β-actin were synthesized (Invitrogen). HPV16 E6 (F: 5′-AGCGACCCAGAAAGTTACCA-3′, R: 5′-GCATAAATCCCGAAAAGCAA-3′), HPV16 E7(F: 5′-CAGCTCAGAGGAGGAGGATG-3′, R: 5′-GCACAAC CGAAGCGTAGAGT-3′), PLK2 (F: 5′-CTACGCCGCA AAAATTATTCCTC-3′, R: 5′-TCTTTGTCCTCGAAGT AGTGGT-3′), DGCR8(F: 5′-AGCGTGAGCTTTACCG AGAG-3′, R: 5′-CTACCCCGTCACCAACACTC-3′), β-actin (F: 5′-TGGCACCCAGCACAATGAA-3′, R: 5′-CTAAGTCATAGTCCGCCTAGAAGCA-3′). QPCR primers used for verifying miRNA microarray results were purchased (GeneCopoeia, Guangzhou, China)

### Cell lines and human tissues

HPV16-positive human cervical cancer cell lines, CaSki and SiHa, were obtained from Shanghai Institute of Cell Biology, China Academy of Sciences. SiHa cells was grown in Dulbecco's modified Eagle's medium (DMEM) with 10% fetal bovine serum(FBS) at 37°C and 5% CO_2_. CaSki cells were grown in RPMI1640 medium with 10% FBS at 37°C and 5% CO_2_.

Ten pairs of HPV16 positive cervical cancer tissues and the adjacent normal tissues were collected from department of obstetrics and gynecology, Southern Medical University. Informed consent was obtained from all the patients, and use of these tissues were approved by Southern Medical University ethics committee. Infection of high-risk or low-risk HPV genotypes in the cervical samples were detected using Hybrid Capture II Test (Digene Corporation). HPV16 infection was verified by qPCR. Each tissue was homogenized in 1 ml TRIzol Reagent using liquid nitrogen extraction, and collected in 1.5 ml Eppendorf tube. The isolated RNA was dissolved in RNase-free water and stored at 70°C.

### Transfection experiments

CaSki or SiHa cells were seeded in six-well plate at 1×10^6^ cells/well and cultured overnight. After washed three times with PBS, cells were transfected with siRNA-e6/e7, si-plk2, si-dgcr8, miR-27b mimics, miR27b inhibitors or control at a final concentration of 100nM using Lipofectamine RNAiMaX (Invitrogen), or transfected with 5 μg pEGFP-e6, pEGFP-e7, pReceiver-M98-plk2, pReceiver-M98-dgcr8 or negative control using Lipofectamine2000 (Invitrogen). Six hours later, the medium was replaced with complete medium which containing 10% FBS. Cell were harvested after 48hours of incubation.

### MiRNA array hybridization, scanning and analysis

Total RNA from 6 samples (three E6/E7 silenced Caski samples and three negative controls) was harvested using TRIzol (Invitrogen) and miRNeasy mini kit (QIAGEN) according to manufacturer's instructions. RNA quantity was measured using the NanoDrop 1000. The samples were then labeled using the miRCURY™ Hy3™/Hy5™ Power labeling kit and hybridized on the miRCURY™ LNA Array (v.18.0). Following the washing steps the slides were scanned using the Axon GenePix 4000B microarray scanner. Scanned images were then imported into GenePix Pro 6.0 software (Axon) for grid alignment and data extraction. Replicated miRNAs were averaged and miRNAs with intensities≥30 in all samples were chosen for calculating the normalization factor. Expressed data were normalized using the median normalization. After normalization, significantly differentially expressed miRNAs were identified through Volcano Plot filtering. Finally, hierarchical clustering was performed to show distinguishable miRNA expression profiling among samples.

### Quantitative real-time PCR

Total RNA was extracted from cells or the tissue samples using Trizol (Invitrogen), and cDNA was generated using the PrimeScriptVR RT reagent Kit (Takara, Dalian, China) or All-in-One miRNA qRT-PCR detection kit (GeneCopoeia, Guangzhou, China). A quantitative real-time polymerase chain reaction (qRT-PCR) assay was performed to evaluate mature miRNAs and mRNA expression using the SYBR Premix Ex Taq (TaKaRa). The relative expression levels of each sample were measured using the 2^−ΔΔ CT^ method with β-actin and U6 snRNA serving as endogenous controls for qPCR of mRNA and miRNA, respectively. Amplifications were carried out according to the manufacturer's instructions in triplicate.

### Western blot

Total protein (50 μg) from samples was separated by 12% SDS-PAGE and transferred to 0.22 micrometer Immobilon-P membranes(Millipore). The membranes were incubated overnight at 4°C with anti-HPV16 E7(Santa Cruz) diluted at 1:500, anti-human p53(Millipore) diluted at 1:1000, anti-human PLK2(Proteintech) diluted at 1:1000, anti-human DGCR8(Proteintech) diluted at 1:1000 or β-actin(Cell Signaling) diluted at 1:1000. After washed with PBST for three times, the membranes were incubated with horseradish peroxidase (HRP)-conjugated anti-mouse IgG (Millipore) diluted at 1:5000 for two hours. HRP activity was detected with SuperSignal West Pico enhanced chemiluminescent (ECL) substrate (Thermo Scientific).

### CCK-8 cell proliferation and activity detection

One hundred microliters of cell suspension (5000 cells/well) were dispensed in a 96-well plate and pre-incubated for 24 hours in a humidified incubator at 37°C, 5% CO_2_, then CCK-8 solution (10 μl) was added to each well of the plate. After 4 hours, the absorbance was measured at 450 nm using a microplate reader. In cytotoxicity assay, paclitaxel (10 μl) was added at a final concentration of 1 μM, cisplatin was added at final concentration of 2 μM in 24 hours after si-contr(or si-plk2) were transfected into cells. The plate was incubated for 0, 24, 48, 72 or 96 hours before the CCK-8 solution was added,

### Transwell assay

Matrigel was thawed at 4°C and diluted with no serum medium at 1:5 and kept on ice. 100 μl diluted matrigel was added to each 8.0 μM transwell chamber and incubated at 37°C. After 4 hours, 200 μl cell suspension was added to the upper chamber of each coated transwell insert and 500 μl RPMI1640 containing 10% FBS was added to the bottom well of the transwell chamber. After the plate was incubated at 37°C for 24 hours, the cells were removed from the upper portion of the transwell filters with cotton swabs. 0.1% Crystal Violet stain (500 μl) was added and incubated for 0.5 hour at room temperature. The stained transwell inserts were washed with PBS for twice, and inverted to allow them to air dry. The ratio of invaded cells was quantified by counting the cells that have invaded onto the lower surface of the porous membrane.

### Apoptosis assay

The caspase-3(Cysteine-requiring Aspartate Protease), which was a key enzyme in the process of apoptosis, was detected to reflect the level of apoptosis cells. Cells were seeded in six-well plate at 1× 10^6^ cells/well and cultured overnight, then transfected with miR-27b mimics, inhibitors, pReceiver-M98-plk2, si-plk2 or controls for 24 hours. Paclitaxel was added in each well at a final concentration of 3 μM. After 24 hours, cells were digested with trypsin and resuspended in lysis buffer on ice for 15 minutes, then the samples were centrifuged at 4°C, 16,000 × g to harvest the supernatant. The total protein concentration of the supernatant was detected with Bradford method. 30 μg total protein of each sample was added to the reaction system and was incubated at 37°C for 60 minutes. The absorbance was measured at 405nm using a microplate reader to calculate the relative amount of caspase-3 in order to indicate the apoptosis.

### Luciferase reporter assay

Wild type or the mutant of whole potential seed regions of human PLK2 mRNA 3′UTR was cloned into vector pEZX-MT05 which contains secrete gaussia luciferase (Gluc) reporter gene. HEK293T cells were seeded into 6-well plates and cotransfected with 3′UTR vectors and miR-27b mimics or negative control. The supernatants were collected 48 hours later, Gluc and SEAP (secreted alkaline phosphatase, the internal control) luciferase activities were measured, and the Gluc/SEAP ratio was used to compare the relative activity of each group.

### Statistical analysis

The data are representative of three independent experiments, and the GraphPad Prism 6 was used for analysis. The data are presented as the mean ± standard deviation and were compared using Student's t-test or one-way ANOVA test, as appropriate. Statistical significance was defined as *P* <0.05.

## SUPPLEMENTARY FIGURE AND TABLE


