# Supplementary material for: MicroRNA-27b up-regulated by human papillomavirus 16 E7 promotes proliferation and suppresses apoptosis by targeting polo-like kinase2 in cervical cancer
Source: Oncotarget. 2016 Feb 20;7(15):19666–79. doi: 10.18632/oncotarget.7531 (PMC4991410; doi:10.18632/oncotarget.7531)
Supplement: Supplementary file 1 [file oncotarget-07-19666-s001.pdf]

## SUPPLEMENTARY FIGURE AND TABLE

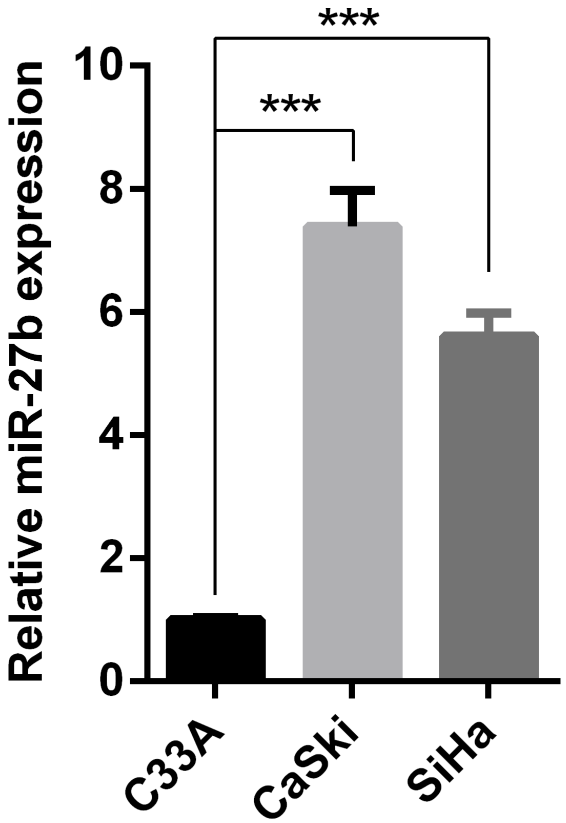


**Supplementary Figure S1: The basal level of miR-27b in C33A, CaSki and SiHa cells.** The basal level of miR-27b in C33A, CaSki and SiHa cells was measured by QPCR. The data are representative of three independent experiments. $^{***}P < 0.001$.

**Supplementary Table S1: Fold changes of differentially expressed miRNAs in microarray**

| Name | Mean of Normalized Intensities (Control) | Mean of Normalized Intensities (Silenced) | Fold Change (Silenced/ Control) |
|---|---|---|---|
| hsa-miR-31-5p | 3.910 | 1.535 | 0.393 |
| hsa-miR-24-3p | 22.151 | 8.242 | 0.372 |
| hsa-miR-103a-3p | 5.653 | 2.108 | 0.373 |
| hsa-miR-27b-3p | 6.288 | 1.888 | 0.300 |
| hsa-let-7b-5p | 3.936 | 1.375 | 0.349 |
| hsa-miR-204-3p | 0.876 | 2.463 | 2.812 |
| hsa-miR-519e-5p | 0.092 | 1.040 | 11.254 |
| hsa-miR-99b-3p | 0.080 | 0.298 | 3.707 |
| hsa-miR-30e-5p | 1.721 | 0.613 | 0.356 |
| hsa-miR-10a-5p | 0.667 | 0.264 | 0.395 |
| hsa-miR-424-5p | 4.781 | 2.361 | 0.494 |
| hsa-let-7g-5p | 3.644 | 1.285 | 0.353 |
| hsa-miR-183-5p | 1.722 | 0.815 | 0.473 |
| hsa-miR-767-5p | 14.541 | 6.158 | 0.423 |
| hsa-miR-151a-3p | 0.817 | 0.198 | 0.243 |
| hsa-miR-148b-3p | 1.032 | 0.413 | 0.400 |
| hsa-miR-33a-5p | 4.637 | 1.551 | 0.334 |
| hsa-miR-30c-5p | 3.536 | 1.672 | 0.473 |
| hsa-miR-182-5p | 2.256 | 0.897 | 0.398 |
| hsa-miR-194-5p | 2.440 | 0.749 | 0.307 |
| hsa-miR-31-3p | 1.216 | 0.412 | 0.339 |
| hsa-miR-660-3p | 2.245 | 10.965 | 4.885 |
| hsa-miR-335-5p | 2.026 | 0.734 | 0.362 |
| hsa-miR-342-3p | 2.244 | 0.789 | 0.352 |
| hsa-miR-193a-3p | 8.809 | 3.940 | 0.447 |
| hsa-miR-25-3p | 1.667 | 0.568 | 0.341 |
| hsa-miR-106b-3p | 0.670 | 0.332 | 0.496 |
| hsa-miR-19b-3p | 6.006 | 2.516 | 0.419 |
| hsa-miR-24-2-5p | 0.513 | 0.195 | 0.379 |
| hsa-miR-32-5p | 2.528 | 1.032 | 0.408 |
| hsa-miR-200c-3p | 11.403 | 4.390 | 0.385 |
| hsa-miR-601 | 0.026 | 0.146 | 5.532 |
| hsa-miR-106b-5p | 10.623 | 3.736 | 0.352 |
| hsa-miR-96-5p | 3.389 | 1.158 | 0.342 |

(*Continued*)

| Name | Mean of Normalized Intensities (Control) | Mean of Normalized Intensities (Silenced) | Fold Change (Silenced/ Control) |
|---|---|---|---|
| hsa-miR-151a-5p /hsa-miR-151b | 1.566 | 0.671 | 0.428 |
| hsa-miR-141-3p | 19.711 | 7.830 | 0.397 |
| hsa-miR-429 | 0.889 | 0.331 | 0.373 |
| hsa-miR-93-5p | 8.589 | 2.973 | 0.346 |
| hsa-miR-665 | 0.132 | 0.576 | 4.352 |
| hsa-miR-301a-3p | 4.032 | 1.576 | 0.391 |
| hsa-miR-191-5p | 1.016 | 0.358 | 0.353 |
| hsa-miR-205-5p | 75.046 | 34.764 | 0.463 |
| hsa-miR-22-3p | 12.559 | 4.073 | 0.324 |
| hsa-miR-20a-5p | 7.843 | 3.659 | 0.467 |
